# Homodigital Pedicled Digital Artery Perforator flaps for fingertip reconstruction - a review of flap options

**DOI:** 10.1016/j.jpra.2022.09.004

**Published:** 2022-10-05

**Authors:** Waseem-Ullah Khan, Aswin Appukuttan, Charles Yuen Yung Loh

**Affiliations:** St Andrew's Centre for Plastic Surgery and Burns, Broomfield Hospital, Court Road, Chelmsford, Essex, United Kingdom, CM1 7ET

**Keywords:** Digital artery perforator flap, DAP flap, Homodigital perforator flap, Islanded digital artery perforator flap, Fingertip reconstruction

## Abstract

**Background:**

Digital artery perforator flaps provide a useful option for reconstruction of soft tissue defects of the digits, and many variations of these flaps have been described for fingertip defects. This review aims to summarize various pedicled digital artery perforator flap anatomical configurations described in literature for fingertip reconstruction and their results.

**Methods:**

A literature review of PubMed, Medline and Embase databases was conducted. Studies reporting homodigital digital artery perforator flaps for fingertip reconstruction without sacrificing the proper digital artery were included in the review. Data collected included flap design, perforator location, pathology, defect location, flap dimensions, flap survival, sensory recovery, range of movement, outcome measures, and complications. The flaps were analysed and categorized based on flap location, flap design, transfer method, and flap innervation.

**Results:**

Pedicled digital artery perforators have been described in various configurations by different authors including propeller, bilobed, V-Y advancement, rotation and turnover flaps. Variations to make these flaps sensate have also been described. According to the available reports, these flaps have low complications and high patient satisfaction rates.

**Conclusion:**

Pedicled digital artery perforator flaps provide a versatile option for the reconstruction of fingertip and thumb tip defects. Various flap options are available for use depending on the reconstructive need. They are quicker and easier to perform than free flaps and can provide excellent outcomes in suitable cases.

## Introduction

Ideal reconstruction of fingertip defects should be with ‘like for like’ glabrous skin, provide padding over the distal phalanx, be sensate and be aesthetically pleasing. The defect size and location, the patient's needs and the surgeon's preference influence the choice of reconstruction. The ideal flap must be simple to perform, allow a one-stage reconstruction with minimal donor site morbidity and have a low incidence of complications. Reconstructive techniques of varying complexity have been described for fingertip defects ranging from skin grafts to free flaps. Among various flap techniques, homodigital flaps limit donor site morbidity to the same digit and provide a single-stage option. Traditionally, the Atasoy^1^ or Tranquilli-Leali^2^ V-Y flap, Kutler flap,^3^ Venkataswami flap,^4^ Segmuller flap^5^ and reverse islanded digital artery flaps^6^^,^^7^ have been the homodigital options for fingertip reconstruction with many others also described. With advances in the understanding of the perforator anatomy, the use of digital artery perforator (DAP) flaps has become vital in the reconstructive armamentarium. DAP flaps have the benefits of traditional homodigital advancement flaps but also offer additional flap configurations for use without sacrificing the digital artery and with comparatively less donor site morbidity. These flaps are easy to perform, do not require microsurgical anastomosis and can be used in multi-digit injuries whilst protecting the neighbouring fingers. Various techniques and modifications of DAP flaps are described in literature. In this review, we aim to summarize the various pedicled homodigital DAP flap options described for fingertip reconstruction.

## Materials and Methods

### Search Methods

A list of MeSH terms was used to search PubMed, Medline and Embase databases in January 2022. No time limits were applied. Keywords such as ‘digital artery perforator flap’, ‘DAP flap’, ‘Homodigital perforator flap’, ‘Islanded digital artery perforator flap’ and ‘fingertip reconstruction’ were used. The search was performed by three independent reviewers.

### Selection criteria

All clinical studies of any evidence level that reported homodigital DAP flaps for fingertip reconstruction, without sacrificing the proper digital artery, were eligible for inclusion. Articles based on non-DAP flaps, heterodigital flaps and non-fingertip reconstructions were excluded. Further relevant articles were identified from the references of published articles. After exclusion of duplicates, two authors independently reviewed the titles and abstracts of the selected studies in the first selection phase. Subsequently, the full texts of potentially eligible studies were reviewed. Any disagreement was resolved through discussion or, if required, after consulting the third author. A data collection form was designed and independently implemented by two authors to allow extraction, manipulation, and comparison of relevant data from the included studies.

### Data extraction and analysis

**Figure 1:**
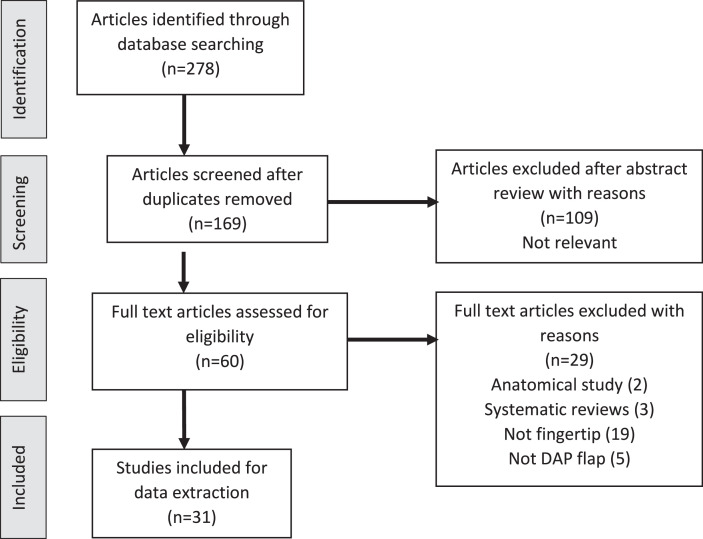
Study selection process flow chart (from the preferred reporting items for systematic reviews and meta-analysis (PRISMA) statement).

Using the selection and exclusion criteria and after eliminating duplicates, 31 articles were found to be eligible for inclusion in the study (Figure 1). All included studies were level 4 on the Oxford Level of Evidence Scale. Data collected included flap design, perforator location, cause of injury, defect location, flap dimensions, flap survival, sensory recovery, range of movement, outcome measures and complications. Descriptive statistics were used to summarize the data. The summary of findings from the included studies is presented in Table 1. There were 495 DAP flaps performed in 453 patients that were used for fingertip reconstruction, which were analysed and categorized. The data from each article included were compiled in a table. Two reviewers read through the data available in each article and ensured that the data were accurate and complete.

**Table 1:** Overview of data extracted from the included studies - patient demographics, flap characteristics and outcomes.

Year	Author(s)	Technique	Patients(n) / Flaps (n)	Age(years)	Cause of defect	Defectsite	Flapsize (mm)	Flaps	ROM (deg)	SWM(g)	S2DP (mm)	Follow up (months)	PROMS	RTW	Complications	ColdInt
1994	Bene et al^30^	Reverse dorsal digital island flap- FTSG to donor site	7(7M)10 fingertip recon (12 flaps in 9 pts in total)	7-52	3 Sharp, 3 crush, 1 other	5IF, 3MF, 2RF		Reverse dorsal digital island flap				17.3 (13 to 25)			All survived, 1 marginal necrosis, 2 nail residue	
1995	Pelissier et al^25^	Reverse dorsal DAP flap- transposition/ Propeller, Donor site -10 primary closure, 7 STSG	17 / 17 fingertip, (total 27)		Work related trauma	5IF, 6MF, 2RF, 4LF	14 × 16	Reverse dorsal digital island flap				>6		8wks (6-9) if fracture fixation and tendon repair) 4 wks	No complications	
1997	Shibu et al^16^	DAP propeller/transposition	7(5M:2F) / 7	36.7 (15-70)	5 crush, 2 sharp	1IF, 2MF, 3RF, 1LF	20 × 15	7 DAP propellar	0-54.2 1 pt 20^o^extensor lag		10	21.5 (16-24)			All survived, 1 partial necrosis, 1 extensor lag treated with splint and physio	2
2001	Koshima et al^11^ presented 2001, published 2006.	DAP flap island propeller flaps - lateral perforators -propeller (VDPF as per Kawakatsu)	5 (4M:1F) / 5	24-48	2 crush, others unclear	2 fingertip, 1 DIP flexion crease, not clear in 2	20 × 7 to 40 × 20	5 DAP propeller		3.61 (only 1 pt)				8 wks	All flaps survived	0
2001	Kim et al^43^	Volar DAP flap - perforators from transverse palmar arch	23(16M:7F) / 25	32.5 (17-65)	All trauma, 18 primary, 5 secondary necrosis	12 IF, 9 MF, 3 RF, 1 LF	27 × 17 (17 × 8 to 40 × 22)	25 Volar DAP propeller			4.2(3-8)	11.5 (6-22)			All flaps survived. Donor site graft loss 2.	
2003	Laoulakos et al^31^	Dorsal reverse adipofascial turn over, bipedicled, SGG to distal flap	9(8m:1F) / 9	41.3(19- 72)	6 Sharp, 3 Crush	4IF, 4MF, 1RF		9 Adipo- fascial turn over			8	6-30			No complications	0
2005	Li et al^32^	IDAP. Dorsal perforator - 2 dorsal digital nerve branches coapted to both proper digital nerve stumps	3 (3M:0F) / 3	17, 22, 25	2 crush, 1 sharp	2 MF, 1 IF (pulp defects)	18 × 16 (17 × 12 to 20 × 18)	3 IDAP			4,4 & 6	Mean 7.3 (6-9)	Satisfied		All flaps survived.	
2006	Takeishi et Al^35^	Innervated reverse dorsal digital island flap, FTSG to cover donor site and bulky pedicles	8(8M)/8	30-47 (mean 37.3)		1T, 2IF, 4MF,1LF	10 × 15 to 22 × 30	8 IRDAP		0.272 (0.036-0.745)	4 (3-5)	Mean 10 (5-19)			All survived. 3 flap congestion &1/3 with partial epithelial necrosis – all revised for bulky pedicle, 1 compression necrosis – requiring skin graft	0
2009	Mitsunaga et al^15^	6 adipo-cutaneous, 3 adipose only, 2 supercharged, 2 extended). Lateral DAP. CT imaging preop. Propeller.	11 (9M:2F) / 13	39 (4-74)	traumatic amputation	1 thumb, 2 IF, 5 MF, 2 RF, 3LF	mean 325 (144 to 800) mm^2^	13 DAP propeller (2 included dig nerve branch)		3.61-4.08		6			All flaps survived, 2 cases of partial skin necrosis (one required debridement and skin graft),	0
2009	Xianyu et al^33^	IDAP laterodorsal fasciocutaneous – dorsal perforators. Dorsal branch of digital nerve coapted to proper digital nerve stump	7 (4M:3F) / 7	52 (36-59)	3 cutting, 1 avulsion and 3 crushing	2 LMF, 1 LIF, 1 LRF, 1 R thumb, 1 RIF, 1 RMF	15 × 12 – 25 × 17 (mean 18.43 × 15.28)	7 IDAP			4.5(4-6)	12 (10 - 36)	2 satisfactory, 2 good, 3 very good	4 wks (3 - 8)	All survived	0
2010	Kawakatsu et al^28^	Dorsal perforator flap. Rotation flap	1 / 1	20	heat press injury	RIF (dorsal DIP crease)	15 × 12	1 dorsal rotation				6	patient satisfied		No complications	0
		Dorsal perforator. V-Y flap	1 / 1	53	mucous cyst	RLF (dorsal DIP crease)	20 × 10	1 dorsal V-Y				12			No complications	0
		Dorsal perforator. V-Y flap	1 / 1	59	mucous cyst	RIF (dorsal DIP crease)	20 × 10	1 dorsal V-Y				6			No complications	0
2011	Suzuki et al^19^ (case report)	DAP propeller flap - dorsal perforator, skin graft to donor	1F / 1	40	after melanoma resection	LIF fingertip	35 × 19	1 DAP	Extensor lag		8.5	4.5			No complications	0
2012	Qin et al^24^	DAP propeller	3(2M:1F)/4	41.3 (37-45)	Trauma	2MF,2RF	20 × 45	4DAP propellar				4-6			Minimal donor site pigmentation and scar contracture	
2013	Ozcanli et al^36^	IDAP flap - included in 2015 article- lateral perforator	17 (15M:2F) / 17	36 (19- 65)	3 sharp, 14 crush	12 right, 5 left. 9MF, 5 IF, 3 RF,	20 × 10 to 35 × 20	17 IDAP		3.22-3.84	3.4 (2 - 4)	7 (6 - 10)			All flaps survived, 2 nail deformities, 1 mild hypersensitivity	1
2013	Basat et al^13^	DAP flap - lateral perforator, propeller.	5 (4M:1F) / 7	27.5(16-60)	3 industrial, 2 domestic accidents	3 left, 2 right. 2 IF, 2 RF, 2 MF, 1 LF		7 DAP propeller.		3.22-4.31		32 (6 -72)			All survived, 1 partial necrosis -debridement and healing by secondary.	0
2015	Ozcanli et al^14^ (AOTT)	DAP propeller (5 had branch of digital nerve) (July 2007 to Feb 2012 15 patients) - lateral perforator	15 (13M:2F) / 15	33 (19-56)	all traumatic amputation	5 MF, 4 RF, 3 IF, 2 LF, 1 thumb	20 × 10 to 20 × 15	10 DAP, 5 IDAP		3.61-4.56	5.3 (4 – 8)	22 (7 - 62)		39 (30-45) days	Transient venous congestion 12	1
2015	Ozcanli et al^37^ (JHS)	IDAP propeller flap (Aug 2011 to May 2014, 65 fingers, 59 patients, only 55 included in study). Lateral perforator.	55 / 61	35 +/- 14 (16 - 65)	52 crush, 3 postop necrosis	37 Rt, 24 Lt, 49 single, 6 multiple fingers. 27 MF, 18 IF, 13 RF, 2 LF, 1 thumb	16 × 7 to 35 × 20	61 IDAP		0.07-1.4	3.5 (2-6)	18 +/- 9 (6 - 36)	3satisfied, 52 highly satisfied		All survived, 2 nail deform, 5 transient venous cong, 2 mild hypersensitivity, 2 pigmentation, 10 deg of extension loss DIPJ in 1.	4
2015	Shen et al^34^	IDAP propeller - distal middle phalanx dorsal perforator; flap paddle over dorsum of P2	10 (6M:4F) / 12	35 (19 - 48)	3 sharp inj, 4 avulsion, 3 crush	All lateral oblique defects; 3 IF, 5 MF, 4 RF	25 × 15 to 30 × 20	12 IDAP	DIPJ 30-60 degrees		5 (4 –6)	8 (8 - 12)			All flaps survived. Transient venous congestion 1.	0
2016	Kostopoulos et al^12^	7 V-Y - dorsal perforator	7 (only 7/15 tip) / 7	55 +/- 17.1		4 right hand, 4 left hand		7 DAP							All survived. 1 wound dehiscence healed by secondary intention.	
2017	Feng et al^18^	DAP propeller flap - lateral perforators	23 (16M:7F) / 31	5.3 (3-12)	9 crush, 5 gouging, 4 twist, 4 burns	12 IF, 9 MF, 7 RF, 3 LF	13 × 12 to 22 × 16	31 DAP			5.1 (4.5-6)	13.8 (6-20)	21 very satisfied and 2 satisfied		All flaps survived.	0
2018	Gulec et al^40^	IDAP propeller – 15. Lateral perforators	15 (14M:1F) / 15	29.27 +/- 10.12		9 IF, 4 MF, 1 RF, 1 LF		15 IDAP	IDAP: DIP flex 84 (+/- 9.67) | ext full		4.87 +/- 1.88	11.8 +/- 8.75	Quick dash (17.26 +/- 12.66)	5.73 +/- 2.25 wks	All survived, marginal necrosis 1 (re-inset with complete survival); neuropathic pain 1; transient venous cong 3.	0
2018	Liu et al^26^	DAP propeller / transposition, (reverse latrodorsal) FTSG to donor site	6(6M) / 7	37.7(23-50)	4 Saw, 2 crush	5MF,2RF	14 × 24 (12 × 20 to15 × 30)	7 DAP propeller / transposition	DIPJ, PIPJ same as opposite side			8(4-15)		4.3(3-6)	No complications	
2018	Losco et al^23^	DADAP, Reverse single pedicle (DIPJ) adipofascial flap with silver dressings for healing by II^O^ intention (17-21 days)	14(12M:2F) / 15	44 (22-63)	15 Sharp	5IF,5MF, 4RF,1LF	18 × 20	15 DADAP	DIPJ 71(65-80) PIPJ 89(80-100)		4.5(3-8)	12	Quick dash2.6(0-9.1) VAS pain0.7(0-4) Aesthetic - 1fair, 2 good, excellent 12	4.4 (4-5)	All survived, 1 partial flap necrosis, 1 prolonged healing epithelialisation (32 days)	
2019	Matei et al^17^	DAP. Lateral perforators. 47 propeller (DAPP), 10 island transposition (DATP), 24 bi-lobed perforator flaps (BLP)	80 / 81					47 DAPP, 10 DATP, 24 BLP			4-14		67 highly satisfied, 12 satisfied, 2 unsatisfied		Venous congestion 7, Epidermolysis 4, superficial necrosis 3.	
2019	Hu et al^20^	Propeller DAP flap - dorsal perforators	10 / 10		7 crush, 1 twist, 1 cut, 1 scratch	6 thumb, 4 fingers, 4 left hand, 6 right hand		10 DAP propeller	IPJ/MCPJ 149 +/- 12.9		8.06 +/- 1.75	3-12			All flaps survived. Transient venous congestion 2; mild pigmentation 3.	0
2019	Appukuttan et al^29^	DAP Unilateral V-Y advancement flap - lateral perforator	10 (6M:4F) / 10	26.5 (1- 52)	9 crush, 1 sharp	3 IF, 3MF, 4LF	14 to 80 mm^2^	10 DAP Unilateral V-Y			3-4 mm (7 pts)	3 (1-8)	7 satisfied, 1 not (nail contour)		All survived.	
2020	Qin et al^21^	Modified dorsolateral proximal phalangeal island flaps	16(14M:2F) / 16	48.8 (30-57)	16 crush, 3 sharp, 8 avulsions	7 IF, 8 MF, 10 RF, 2 LF	15 × 13 to 27 × 20	16 MDPP	DIPJ 74.8 +/- 6.2		8.0 +/- 1	8.7 (5 -12)	MHQ 85 +/- 3.7	6.3 +/- 1.2 wks	13 full survival, 2 transient venous congestion, 1 partial distal loss due to congestion	0
		Homodigital dorsal DAP flaps	11 (9M:2F) / 11	49.3 (39-58)			18 × 14 to 25 × 20	11 Dorsal DAP	DIPJ 75 +/- 4.6		8.5 +/-1.3	8.4 (6-13)	MHQ 85.9 +/- 3	6.5 +/- 1.1 wks	9 survived fully, 2 venous congestion with flap survival	
2020	Ayhan et al^39^	(IDAP flap) island flap as per Ozcanli technique – lateral perforators	15(14M:1F) 17/21	47.2+/-12.9 (26-62)	3 saw, 14 crush			17 IDAP	DIPJ 77.3+/- 3.5 (70-80)	2.83- 4.93	6.4 (3- 10)	13.8 (7-18)	9 satisfied, 8 Highly satisfied	22.5 (15-30) Days	All survived, Superficial necrosis 3 (2 patients) healed secondarily with dressings	0
2020	Dionyssiou et al^22^	DADAP- adipofacial with SSG (rotation advancement) (dorsal defect + extended to fingertip) propeller -9, turn over -3	8 (7M:1F) 12	17-56	Crush, avulsion, clean cut	3IF, 6MF, 2RF,1LF	25 × 15 to 80 × 30 (extended –fingertip)	12 DADAP (rotation advancement)				18 (6-36)			All survived, 1x distal tip necrosis, 3x delayed healing with haematoma & partial SSG failure	0
2021	Ehya et al^27^	V-Y modification	21(15M:6F) /24	32.5 (18-55)	3 Sharp, 13 twist, 5 crush	5IF, 8MF, 10RF, 1LF	11 × 14 to 27 × 20	24 DAP V-Y modification	DIP 0-60		6.80+/- 1.14 (4.5-9.4)	4.33(3-6)			All survived, 5 Tension blisters and swelling, 2/5 venous congestion and partial necrosis – recovered by suture removal, vasodilators and dressings	0
2021	Cavit et al^38^	IDAP	83(70M:13F)/ 93(85-acute and 8-late)	35.2 (5-65)	11 Sharp, 74 Crush	12T, 23IF, 38MF, 16RF, 4LF Transverse -57, Volar oblique -17, Lateral oblique- 9, Dorsal oblique -7, Pulp defects -3	16mmx7mm to 40mmx 20mm	IDAP	Extensor lag 20^o^ IPJ thumb and 10^o^ 15^o^ DIPJ IF	2.44 -4.56	3.71+/-0.97mm (2-6)	33.1 (12-62)	75 highly satisfied, 8 satisfied		All survived, 4 Hyperesthesia, 6 venous congestion corrected by elevation and loosening of dressings, 4 epidermolysis healed primarily, 2 mild hyperpigmentation	18 (mild)

Abbreviations: DAPP – Digital artery perforator propeller, IRDAP – Innervated Reverse Digital Artery Perforator, IDAPP – Innervated digital artery perforator propeller, DATP- Digital artery perforator transposition propeller, BLP – Bilobed perforator propeller, DADAP – Dorsal adipofacial digital artery perforator, PPDAP – Predictable pattern Digital Artery Perforator, ROM- Range of movement, SWM- Semmes Weinstein Monofilament, S2PD- Static 2 Point Discrimination, PROM- Patient reported outcome measures, RTW- Return to work, Cold.Int- Cold intolerance

## Results

### Anatomical characteristics of Digital Artery Perforators

The arterial anatomy of the digits was studied in detail by Strauch et al.^8^ They identified three palmar arches in the digits arising from the proper digital arteries: the proximal and middle arches at the level of the C1 and C3 cruciate pulleys (lying deep to the flexor tendons) and the distal arch just distal to the profundus tendon insertion as a convergence of the terminal digital arteries. The dorsal branches were arranged in a repetitive pattern of condylar, metaphyseal and dorsal skin vessels in the proximal, middle and distal phalanges.

The term ‘digital artery perforator’ was coined by Koshima et al.^11^ and the term ‘DAP flap’ was used to describe flaps based on small perforators (arterioles and venules) arising from the digital arteries which were found to perforate the thin fascia and adipose tissue and hence considered as perforators rather than branches. The venules that drain the skin connect to the dorsal and volar subcutaneous venous systems. They found an abundance of arterioles and venules between these perforators in the mid lateral line of the digits. The dorsal arterial and venous blood supply to the finger lie in the subcutaneous tissue immediately subjacent to the dermis and superficial to the level of the paratenon of the extensor apparatus. Strauch et al and Braga Silva et al.^8^^,^^9^ showed that the dorsal digital artery perforator branches occur at constant and predictable intervals from the proximal interphalangeal joint, and Dellia et al.^10^ showed that the arrangement of those perforators is constant extending from 5-15 mm proximal to the eponychial fold.

Kostopoulos et al.^12^ described the vascular cutaneo-tendino-osseous complexes (VCTOC) which originate from dorsal perforators from the proper digital arteries and vascularize all tissues of the digit, not just the extensor apparatus. They identified rich connections between the subdermal vascular networks from the dorsal perforator branches and also noted that the dorsal perforators have a ‘predictable pattern’ of location from the digital joints^12^.

### Versatility and configurations of DAP flaps for fingertip reconstruction

**Figure 2:**
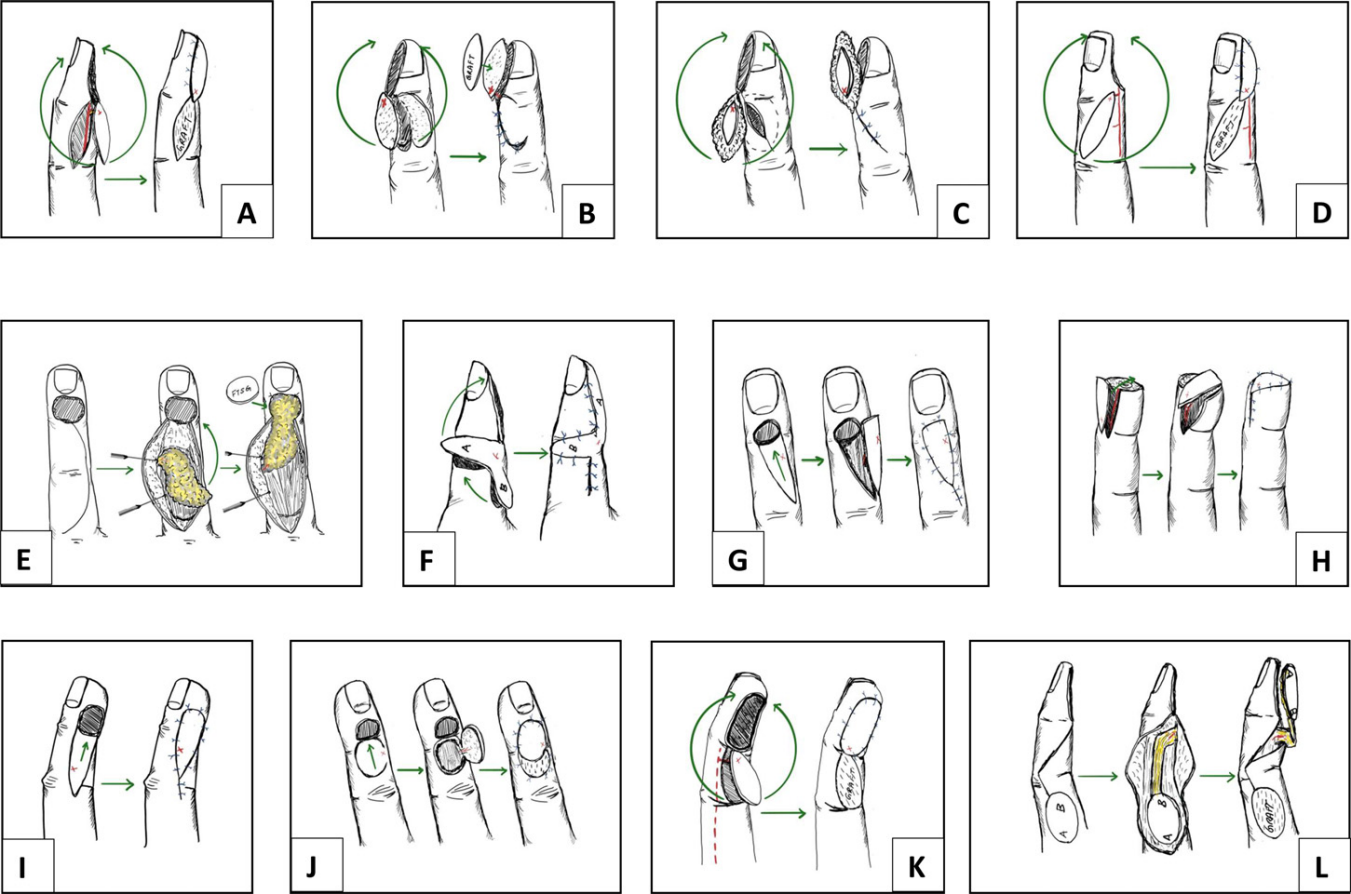
Illustration of various DAP flap configurations identified from review showing potential arc of rotation. (A) Lateral DAP propeller flap. (B) Adiposal only DAP propeller flap. (C) Adipo-cutaneous DAP propeller flap. (D) Dorsal DAP propeller flap. (E) Dorsal Adipofascial Digital Artery Perforator (DADAP) flap (F) Bilobed DAP flap. (G) Dorsal V-Y DAP flap. (H) Lateral V-Y DAP flap for transverse tip amputations. (I) Lateral V-Y DAP flap for distal lateral defects. (J) Dorsal rotation DAP flap. (K) Volar DAP flap. (L) Modified dorsolateral proximal phalangeal (MDPP) island flap.

Digital Artery Perforator (DAP) flaps were categorised into various configurations and modifications depending on the flap location, flap design and transfer method. These are described below, and the configurations are illustrated in Figure 2.

1. DAP Propeller (DAPP) flaps:

Koshima et al described the first DAP flaps based on perforators on the mid-lateral aspect of the fingers.^11^ Their original series comprised five propeller flaps oriented along the mid-lateral axis (Figure 2A). The dissection was above the plane of the main neurovascular bundle and the pedicle contained the most favourable or dominant DAP, subcutaneous venules and a small amount of subcutaneous fat. The flaps were used for reconstruction of fingertip and dorsal distal interphalangeal (DIP) defects. Similar flaps were also described by Basat et al.^13^ and Ozcanli et al.^14^ for traumatic tip amputations of the thumb and fingers.

Mitsunaga et al.^15^ described various modifications in addition to the standard adipo-cutaneous flaps including adiposal only DAPP flaps (Figure 2B), supercharged DAPP flaps and extended (adipo-cutaneous) DAPP flaps based on lateral perforators. Indications were thumb and fingertip defects following trauma or for painful amputation stumps. Two flaps were supercharged by anastomosing a proximal flap perforator to a volar DAP vessel as the distal flap circulation was found to be unstable. The extended DAPP flap included adiposal tissue beyond the skin paddle to provide a larger surface area (Figure 2C). This modification is useful when extra soft tissue is required for coverage of the defect, still leaving a smaller donor skin defect.

Shibu et al. described seven islanded transposition flaps for transverse or oblique fingertip amputations.^16^ In their series one flap had partial necrosis and two patients developed cold intolerance but settled over time with conservative management. These patients had recovery of protective sensation and adequate tissue support for straight nail growth. Matei et al.^17^ described 47 propeller and 10 islanded transposition flaps raised on a lateral DAP vessel to cover mainly fingertip defects. Feng et al.^18^ reported on 31 lateral perforator-based DAPP flaps in paediatric patients (mean age 5.3 years, range 3-12 years) for traumatic and burn injuries. The axis of the flap was oblique across the dorsum of the middle phalanx (Figure 2D).

Various authors have described propeller flaps based on dorsal DAP vessels. Suzuki et al.^19^ described a 35 × 19mm flap raised from the ulnar lateral aspect of the index finger but on a dorsal DAP to cover a dorsal defect with exposed distal phalanx following resection of a melanoma insitu. Hu et al.^20^ described 10 DAPP flaps based on dorsal perforators for distal thumb and fingertip defects. The series by Qin et al.^21^ had 11 similar dorsal DAP flaps. Dionyssiou et al.^22^ described 12 Dorsal Adipofascial Digital Artery Perforator (DADAP) flaps in eight patients. The DADAP flap is based on the dorsal perforators of the proper digital artery and is harvested as an island flap (Figure 2E). The pedicle is completely dissected in order to improve the arc of rotation for use as a propellar flap or turnover flap. The flap marking is aided by the predictable vascular pattern on the dorsum of the fingers^9^^,^^10^ indicating also the entrance of the perforator into the flap. Whilst approaching the pedicle during dissection a release of Cleland's ligament is indicated to enhance the arc of rotation. It is possible to narrow but not skeletonize the pedicle which should contain the nutrient vessels and a thin fibro-fatty tissue layer. The recipient site adipofascial flap is covered with split thickness skin graft. Consequently, the use of DADAP as described can be safely expanded in a more distal level and is expected to permit the coverage of defects distal to the DIPJ. Losco et al.^23^ described similar rotation turn over flaps in 15 fingertip injuries (ulnar or radial oblique- Allen's type II-III) with bone exposure. The flaps were based on the dorsal branch of the digital artery dissected just proximal to DIPJ. The donor site was closed primarily with the distal end of flap dressed with hyaluronic acid and silver ointment impregnated gauze till complete re-epithelisation (17-21 days; mean 19 days). Qin et al.^24^ described four DAPP flaps raised on a dorsal branch of the digital artery as a pivot point at the mid-point of mid-lateral line of the middle phalanx. The donor site was skin grafted (Figure 2D). Pelissier et al.^25^ described 17 flaps for fingertip reconstruction as propeller flaps based on previously described anatomy.^8^ These flaps are useful for dorsal and volar defects. Liu et al.^26^ described complex nail matrix reconstruction in seven digits with reverse dorsolateral flaps based on DAP vessels with good results and recovery of some protective sensation.

Ehya et al.^27^ performed 24 DAP flaps in 21 patients, which are described as modifications of V-Y flaps but appear to be propeller flaps based on dorsal DAP vessels at the level of the DIP joint (Figure 2D). This flap can be used to repair distal dorsal defects of the fingertip. The donor site was skin grafted.

1. *Bilobed DAP flaps:*

Matei et al.^17^ also described 24 bilobed DAP flaps in addition to other configurations in their series. The bilobed design (with the first transverse/oblique flap and second smaller longitudinal flap) allowed closure of larger defects without a skin graft for the donor site. However, the flaps were not fully islanded in their design and had an intact skin bridge at the base (Figure 2F).

2. *V-Y DAP flaps:*

Kawakatsu et al.^28^ used islanded dorsal V-Y perforator flaps based on a single dorsal DAP vessel from the transverse palmar arch proximal to the DIP joint or arising directly from the digital artery to cover dorsal DIP crease defects (Figure 2G). The perforator was identified as ‘a slightly whitish string like structure’ (under tourniquet) at the lateral border of the extensor tendon.^21^ They used the term ‘Dorsal digital artery perforator flaps (DDPF)’ to describe these flaps. We have previously published a series of 10 unilateral V-Y DAP flaps for reconstruction following traumatic Allen type 3 fingertip amputations.^29^ The flap is similar to a unilateral Kutler flap but raised on a lateral DAP, increasing its mobility and reach, and advanced across the distal phalanx to cover the entire fingertip defect with a single flap. The donor site was closed primarily (Figure 2H). Kostopoulos et al.^12^ also described a larger lateral V-Y DAP flap (Figure 2I)

3. *Rotation DAP flaps:*

Dorsal rotation DAP flaps can be used to cover defects on the dorsum of the distal digit as described by Kawakatsu et al.^28^ They reported on a 15 × 12mm dorsal rotation flap based on a dorsal DAP to cover a DIP extension crease defect (Figure 2J). The flap was from the dorsum of the middle phalanx with a very narrow skin bridge over the perforator pedicle. Skin grafting the donor site may be necessary if more movement of the flap is required to cover the defect.

4. *Modified dorsolateral proximal phalangeal (MDPP) island flaps:*

Qin et al.^21^ described 16 islanded flaps raised from the dorsum of the proximal phalanx, with the pivot point at the middle of the middle phalanx and based on a constant dorsal branch from the proper digital artery (PDA). Though not described as perforator flaps in the article, they appear to be based on dorsal DAP vessels. The pedicle between the pivot point and the skin paddle consisted of a subcutaneous tissue component around 8 mm wide which was connected to the PDA by a rectangular soft tissue attachment. The flap was turned over with this attachment as a hinge (Figure 2L) to cover pulp defects. According to the authors, the donor site over the proximal phalanx can provide a larger skin paddle than the middle phalanx and has better donor and recipient site aesthetics. Bene et al.^30^ in his series of 10 flaps in nine patients described similar flaps raised from the dorsum of the middle or proximal phalanx as reverse dorsal digital island flaps. The flap pedicle consisted of longitudinal arterial and venous networks of the dorsal adipofascial layer of the fingers with their numerous distal connections with dorsal branches of the proper digital arteries.^8^ The arc of flap rotation could reach up to 180 degrees allowing reconstruction of dorsal defects of fingertip. The donor site was skin grafted. One extended flap had peripheral necrosis due to incorporation of tissue from proximal third of the proximal phalanx.

5. *Predictable Pattern Digital Artery Perforator (PPDAP) flaps:*

Kostopoulos et al.^12^ described seven lateral V-Y DAP flaps for the reconstruction of radial, ulnar, dorsal and volar defects on the distal phalanx following resection of skin, soft tissue or joint lesions. These were based on their concept of Predictable Pattern Digital Artery Perforator flaps. They used data from prior perforator mapping studies for flap planning instead of perforator identification with a doppler. The flap was raised from the radial or ulnar side of the digit. The pedicle was located dorsal to the skin paddle and included a cuff of subcutaneous tissue. The proximal end the V-Y flaps extended as far as the PIP joint line (Figure 2I).

Laoulakos et al.^31^ described dorsal reverse adipofascial flaps for dorsal oblique or transverse fingertip amputations at the level of nail fold in nine patients. The flaps were bipedicled at the level of distal dorsal branches of digital arteries. The germinal matrix of the nail needs to be excised to avoid nail spicules growing back. The donor site skin was closed primarily with a split skin graft. There was no incidence of cold intolerance and overall results were satisfactory.

6. *Innervated digital artery perforator (IDAP) flaps:*

The first series of IDAP flaps appears to be by Li et al. in 2005^32^ although these were not termed as perforator flaps in their article. They reported three pulp and tip reconstructions using flaps raised from the dorsum of the middle phalanx and perfused by a dorsal perforator at the level of the DIP joint which they termed as the ‘End Dorsal Branch of the Digital Artery’ (EDBA). The flaps were called ‘Innervated Reverse Island Flaps’ (IRIF). The proximal flap included two dorsal digital nerve branches that were divided and coapted to the proper digital nerve stumps. Xianyu et al.^33^ described seven homodigital propeller ‘latero-dorsal fascio-cutaneous’ IDAP flaps for fingertip defects based on the dorsal branch of the proper palmar digital artery. These vessels were again not termed as perforators in their article, but rather as dorsal branches. This may be because the term DAP had not come into widespread use at that time. The dorsal digital nerve branches were coapted to the proper digital nerve stumps for innervation. Shen et al.^34^ described 12 IDAP flaps from the dorsum of the middle phalanx and based on a distal middle phalanx dorsal DAP for traumatic lateral oblique defects. The flaps included the contralateral dorsal digital nerve divided proximally during elevation and coapted to the proper digital nerve on the side of the injury and were rotated 90 degree to reach the lateral oblique defect. Ehya et al.^27^ also described IDAP flaps with anastomoses between the dorsal digital nerve branches in the flap to the digital nerve stump in the wound. Takeishi et al.^35^ described innervated reverse dorsal digital island flaps in eight patients based on dorsal branches of digital artery and the dorsal adipofascial arterial network.^8^^,^^30^ The pedicle dissection included a branch of dorsal digital nerve (superficial branch of radial nerve in thumb) or dorsal branches of the proper digital nerve to anastomose with injured digital nerve ends. The flap dissection beyond DIPJ was discouraged as three out of eight of patients developed post op venous congestion for 4-5 days requiring revision for bulky pedicles (Figure 2L).

Ozcanli et al described 93 IDAP flaps for reconstruction of transverse and oblique fingertip amputations in their reports in 2013,^36^ 2015^37^ and 2021^38^ for crush injuries or for postoperative pulp necrosis. The flaps were preferably raised from the ulnar side and extended proximally onto the dorsum of the middle phalanx to increase the length. The pedicle included the DAP, the terminal branch of the digital nerve and the subcutaneous venous system (including a 2-3 mm cuff of subcutaneous tissue). The flaps were rotated 90 to 180 degrees to reach the dorsum, pulp or the tip. Ayhan et al.^39^ recently described their series of IDAP flaps similar to the Ozcanli technique with 17 flaps for palmar oblique or transverse fingertip amputations. Gulec et al.^40^ have also described 14 IDAP propeller flaps incorporating the terminal branch of the digital nerve as in the Ozcanli's series and the series by Cavit et al.^38^ reported on 93 similar IDAP flaps in 83 patients.

Chen et al.^41^ described reconstruction in 114 fingertip and pulp defects using IDAP propeller flaps with an extended pedicle to incorporate the palmar arch just proximal to DIPJ crease and ligation of PDA. Wei et al.^42^ described 31 homodigital anterograde islanded pedicle flaps based on the dorsal perforators in the middle phalanx.^8^ The blood flow is provided by the anterograde flow from the dorsal branches at the level of DIPJ (two to three perforators) but includes proper neurovascular bundle dissected within the flap. Therefore, we have excluded data from these articles;^39^^,^^40^ however, anatomical and surgical description will certainly add to understanding of DAP flaps.

7. *Volar DAP flaps:*

Kim et al.^43^ described the volar digital flap based on the transverse digital palmar arches. This report was before the description of the DAP flaps, but the flaps seem to be supplied by transverse palmar arch perforators. The skin paddle was raised from the volar aspect of the digit proximal to the defect and rotated to provide glabrous skin for fingertip defects (Figure 2K). The donor site was grafted. Flexion contracture of the digit was mentioned as a complication.^43^ These flaps do not seem to have gained popularity as they violate the volar aspect of the finger.

## Discussion

The main purpose in the treatment of fingertip injuries is to obtain a stable, painless, aesthetically pleasing and sensate fingertip. The ideal reconstructive flap should be versatile, reliable, sensate, single-stage, easy to perform, avoid prolonged immobilisation with few complications and have no or minimal donor site morbidity. DAP flaps described above fulfil these criteria but individual surgical techniques used and how easy these are to perform vary according to surgeon's skills and preference based on site, size and cause of defect. Articles included in this review are based on level 4 evidence with small number of patients studied and due to possible subjective interpretation of outcomes we found it difficult to categorise individual techniques based on the ease of performing and overall outcomes.

### Indications for pedicled DAP flaps in fingertip injuries

Pedicled DAP flaps are indicated for smaller defects of the fingertip including dorsal, volar and lateral defects as well as various configurations of tip amputations. The majority of the reports on pedicled DAP flaps for digital tip defects are for the fingers, but many authors have also used it for thumb tip defects.^13^^,^^14^^,^^20^^,^^33^^,^^39^ Indications were mostly traumatic defects, but they have also been applied for other defects including after benign^12^^,^^28^ and malignant^17^^,^^19^ tumour resections and revision of painful amputation stumps.^15^ The flaps are not suitable for large defects. From the review, flap width ranged from 7 to 22 mm and flap length ranged from 13 to 40 mm. Some authors have suggested a delayed approach to DAP flap reconstruction when there is extensive soft tissue damage as the perforators around the distal interphalangeal joint may be rendered unreliable.^44^

### Pre-operative perforator identification

There was variation in the technique of pre-operative identification of the perforators, with many authors using the hand-held doppler preoperatively^5^^,^^11^ and others raising the flap without pre-operative localisation.^12^^,^^14^^,^^15^^,^^23^^,^^24^^,^^25^^,^^29^^,^^30^^,^^31^^,^^35^ Kostopoulos et al.^12^ and Dionyssiou et al.^22^ raised their flaps using mapping data from prior studies (PPDAP flaps) or using the predicable position of the dorsal perforators as mentioned earlier.^8^^,^^9^^,^^10^ Shintani et al.^45^ described the usefulness of colour doppler ultrasonography for the identification of perforators and flap planning. Mitsunaga et al.^15^ described multidimensional CT imaging for preoperative assessment of the perforators, especially in cases with severe injury or scarring. However, pre-operative imaging is not widely used in practice.

### Donor site closure

Donor sites up to 1 cm wide maybe amenable to primary closure.^15^^,^^17^ Wider defects may need grafts to prevent compression of the pedicle.^13^^,^^35^ Skin grafts can be obtained from the amputated part, if available.^15^ Artificial dermis can be used to resurface the donor site and most of these epithelialize without further skin grafting.^15^

### Sensory recovery

A sensate fingertip is important for precise stereognosis, object identification and function. In their study, Cavit et al^38^ concluded that IDAP flaps lead to better sensory recovery compared to published results of other reconstructive options. IDAP flaps larger than 20 mm x10 mm were able to remain sensitive, whereas previous studies have shown that sensory recovery reduces in flaps larger than 10 × 10 mm without neurorrhaphy.^38^ However, from all the available data in our study, we were unable to establish whether there was a definite sensory advantage for IDAP flaps over non-innervated DAP flaps and various authors have also cast doubt on this.^13^^,^^16^^,^^21^^,^^26^^,^^33^^,^^46^^,^^47^. It has also been found that patient satisfaction is not always dependent on sensory recovery.^39^ Using univariate and multivariate logistic regression analyses, Ayhan et al found that sensory recovery was a less important factor for patient satisfaction than cold intolerance that adversely affected patient-reported outcomes.^39^

### Complications

The complications noted from the review were venous congestion, partial necrosis, cold intolerance, hypersensitivity, pin-cushioning, pigmentation, nail deformity and extensor lag. Venous congestion seen in 39 flaps (7.87 %) resolved spontaneously but took up to five to seven days to resolve.^14^^,^^42^ There were 21 partial flap losses (4.2 %) of which sixteen healed secondarily but two needed grafting and one needed revision surgery. Cold intolerance was reported only in 26 flaps (5.2 %) from the review, but Ayhan et al.^39^ reported this as 41.2% in their series with values of 6 to 42% reported in literature.^37^^,^^38^ Cold intolerance improved with time.^16^^,^^39^ The review also identified flap pigmentation (1.4%), hyper-sensitivity (0.7%), nail deformities (0.6%), nail residue (0.4%), extensor lag (0.4%) as rare complications. Pin-cushioning has been mentioned by Mitsunaga et al.^15^ as a common complication but was not mentioned in other studies. All studies reported high patient satisfaction rates.

### Technical tips

DAP flaps are best performed under magnification. If a dominant perforator cannot be found, adipose tissue, which would contain some arterioles is preserved at the base to nourish the flap.^11^^,^^13^ It is important to leave a cuff of subcutaneous tissue around the perforator to preserve venous return. Division of the Cleland and Grayson ligaments will enhance the rotation of the propeller flaps.^16^^,^^26^^,^^35^ Innervated flaps can be performed by including a nerve branch supplying the flap or by dividing and coapting a nerve branch in the flap to a digital nerve stump though it is unclear whether this translates into improved sensation. Venous congestion is transient in most flaps and may take up to a week to resolve. Tight donor site closure should be avoided to prevent venous congestion. Donor sites wider than 1cm may be better covered with a skin graft or artificial dermis.

## Conclusions

A wide range of reconstructive options have been described for fingertip injuries, and all of these have shortcomings and disadvantages including inadequate sensation, donor site morbidity, limited flap size, need for immobilisation, flexion contractures and prolonged times to return to work. The Type of injury, expectations of the patient, surgeon's skill and experience should be considered whilst choosing the most appropriate treatment method. In our opinion, digital artery perforator flap elevation can be challenging for the novice surgeon; however, basic microsurgical dissection skills and a thorough knowledge of the vascular anatomy would help in quick and safe flap dissection. These flaps are a useful option in addition to traditional homodigital, heterodigital and free flaps in the reconstruction of selected thumb and fingertip defects. Various configurations are available for use based on the defect size and location. DAP flaps are associated with low complication rates and can achieve good to excellent functional and aesthetic outcomes.

## Author Contributions Statement

All authors contributed extensively to the work presented in this paper. WK, AA and CYYL conceived the study and were involved in study design, data extraction and analysis. WK and AA contributed as first authors and prepared and edited the manuscript.

## Ethical approval

Not required.

## Conflicts of interest

None declared.
